# Evaluating biosecure entry and exit protocols and education methods in a mock livestock facility

**DOI:** 10.3389/fvets.2026.1724406

**Published:** 2026-02-03

**Authors:** Abby Schuft, Sally Noll, Kevin Janni, Brian Hetchler, Krishona Martinson, Erin Cortus

**Affiliations:** 1 Department of Animal Science, University of Minnesota, Saint Paul, MN, United States; 2 Department of Bioproducts and Biosystems Engineering, University of Minnesota, Saint Paul, MN, United States

**Keywords:** biosecurity, compliance, education, errors, livestock, poultry

## Abstract

**Introduction:**

Teaching biosecure protocols to farm workers and visitors is essential to maintaining healthy herds and flocks in animal agriculture. Our study objective was to evaluate the effects of protocols with varying steps, education methods, and short and mid-term retention on the number of errors and time to complete entry and exit processes in a mock livestock facility.

**Methods:**

University participants were recruited to learn and demonstrate biosecure protocols. Three simulated farm entryways were constructed with a unique set of protocols assigned (Protocol) to each entryway. Using common industry practices, Protocol 1 established three core procedures: signing a logbook, removal of outerwear and personal items, and management of a phone or other device. Protocols 2 and 3 used the same core procedures with 3 and 4 additional steps, respectively. The additional steps included changing footwear, crossing a line of separation, using hand sanitizer, donning barn-specific clothing (Protocol 3 only). Participants learned procedures via one of three educational modalities (Method): listen, read, or watch. Short-term retention was assessed as participants completed all three protocols in forward (Entry) and reverse order (Exit) starting from a randomly assigned initial protocol (Initial). When half of the participants returned after a lapse of time (>1 month; Round), they had to rely on their recall of the procedures. Mixed-effects linear regression was used to model entry and exit time (Time), and a Poisson regression was used to model the entry and exit errors (Errors) committed.

**Results:**

Analysis showed education Method did not influence Errors or Time during Entry or Exit. Participants made 1.37 times more Entry Errors (*P* ≤ 0.02) and 1.19 times more Exit Errors (*P* ≤ 0.17) during round 2 compared to round 1. Compared to Protocol 1, participants made 1.47 times more Errors on average during Protocol 2 (*P* ≤ 0.02) and 1.87 times more during Protocol 3 (*P* ≤ 0.001). The time to complete the entry procedures was associated with Protocol, Round and the Initial experience, but exit time was only associated with Protocol and Round.

## 1 Introduction

Biosecurity is the prevention and reduction of infectious pathogen introduction and spread. In animal agriculture, biosecurity is important to protect the health of animals, the economy, food safety, and in some cases, public health (1–3). Infectious disease in animal agriculture creates economic losses for farms, retailers, and consumers (4). The United States Department of Agriculture (USDA) estimates more than $3.3 billion was spent in the response and recovery of the 2014–2015 highly pathogenic avian influenza outbreak (5). Moreover, preventing or reducing the incidence of disease requires additional expense by a farmer as they invest in biosecurity measures (4, 6). The cost of implementing biosecurity includes capital investments (i.e., fences, wash stations, etc.), consumables such as feed, alternative medicines and treatments, and labor (6), in addition to disinfectants, cleaning supplies and personal protective equipment for animal caretakers. Schreuder et al. (7) concluded that biosecurity compliance improved when the cost to implement was low. However, Preite et al. (3) discovered up to 40% of farmers remained unwilling to participate in biosecurity measures when resources were offered for free or were fully subsidized.

Previous research has shown that implementing biosecure protocols at the entry and exit of an animal facility can be effective at reducing the risk of disease introduction. The farm/barn entry and exit are a high risk setting because of the frequency at which people and items pass through and the proximity to live animals (8). In addition to changing boots, coveralls and handwashing, Anderson et al. (8) further demonstrated a physical barrier at the line of separation (LOS) significantly reduced pathogen movement in a biosecure entry and exit. However, this barrier did not reduce all risk, and the authors suggested further training for employees on the topics of footwear choices and procedures for removing footwear.

Biosecure entries and exits reflect structural and operational biosecurity elements. Structural biosecurity refers to tangible and fixed cost features (9). A physical barrier at the LOS is an example of structural biosecurity. Operational biosecurity relies on the behavior and actions of humans caring for the animals inside and perhaps needs the most attention because it is the least dependable aspect of biosecurity (9, 10). How an animal caretaker performs prescribed entry and exit procedures is an example of operational biosecurity (9).

Racicot et al. (11) documented biosecure protocols for entering and exiting poultry barns were poorly executed in on-farm research conditions and later suggested a lack of understanding as the primary reason (10, 11). On-farm compliance with biosecurity measures continues to be a challenge in the present day (12, 13). To increase understanding of biosecurity protocols and requirements, it seems that additional, or more thorough, training is a starting point.

Biosecurity training and education needs to be constructed carefully to optimize learning for all (14, 15). The adult workforce has differing preferences in how they receive new information when categorized by generation (16). However, without taking age into account, Merrill et al. (16) showed understanding of biosecurity risk improved when it was conveyed graphically rather than linguistically or numerically.

Within the poultry industry specifically, the frequency which a person visits a barn will vary. Farm workers may visit a barn multiple times in 1 day or a week compared with a service provider that may visit only once per quarter. Our review found gaps in documented knowledge of training methods and compliance of biosecurity within these two groups (farm workers and service providers), especially those that do not practice biosecurity frequently.

Our study objective was to evaluate the effects of protocols with varying steps, education methods, and short and mid-term retention on the number of errors and time to complete entry and exit processes in a mock livestock facility. Our hypothesis was that the education method would influence the number of entry and exit errors and the time to complete prescribed protocols. We also hypothesized that the number of steps needed to complete the prescribed protocols would influence the number of entry and exit errors. Additionally, we assumed a lapse in time between visits would increase errors and time used to complete the procedures.

## 2 Materials and methods

The protocol for human participation and observation as human subjects research was deemed exempt by the University of Minnesota Institutional Review Board (IRB STUDY00012048).

### 2.1 Experimental setting

**Figure 1:**
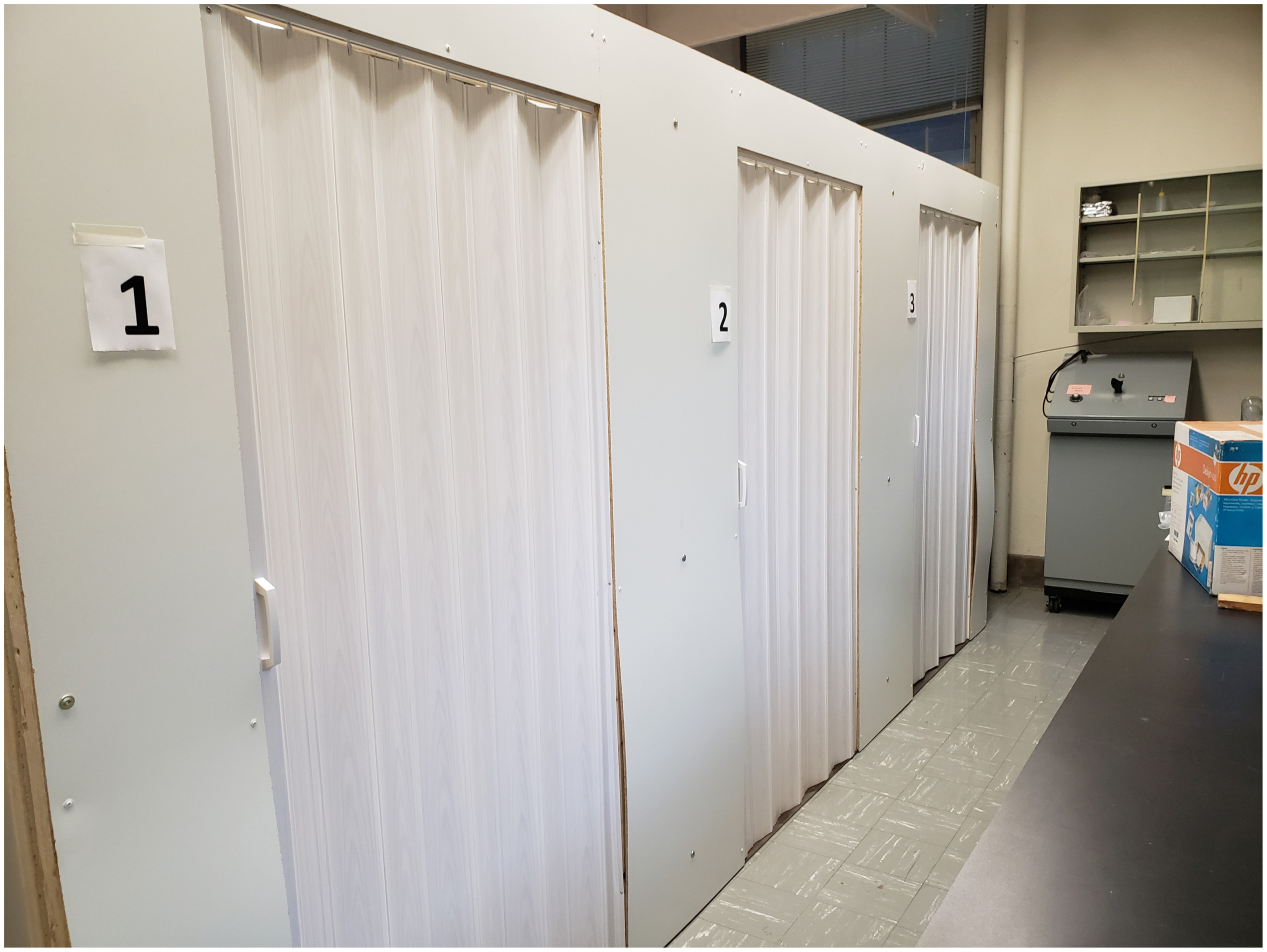
Three simulation spaces for barn entry and exit protocols were constructed inside a laboratory space at the University of Minnesota. The simulated exterior doors are shown.

**Figure 2:**
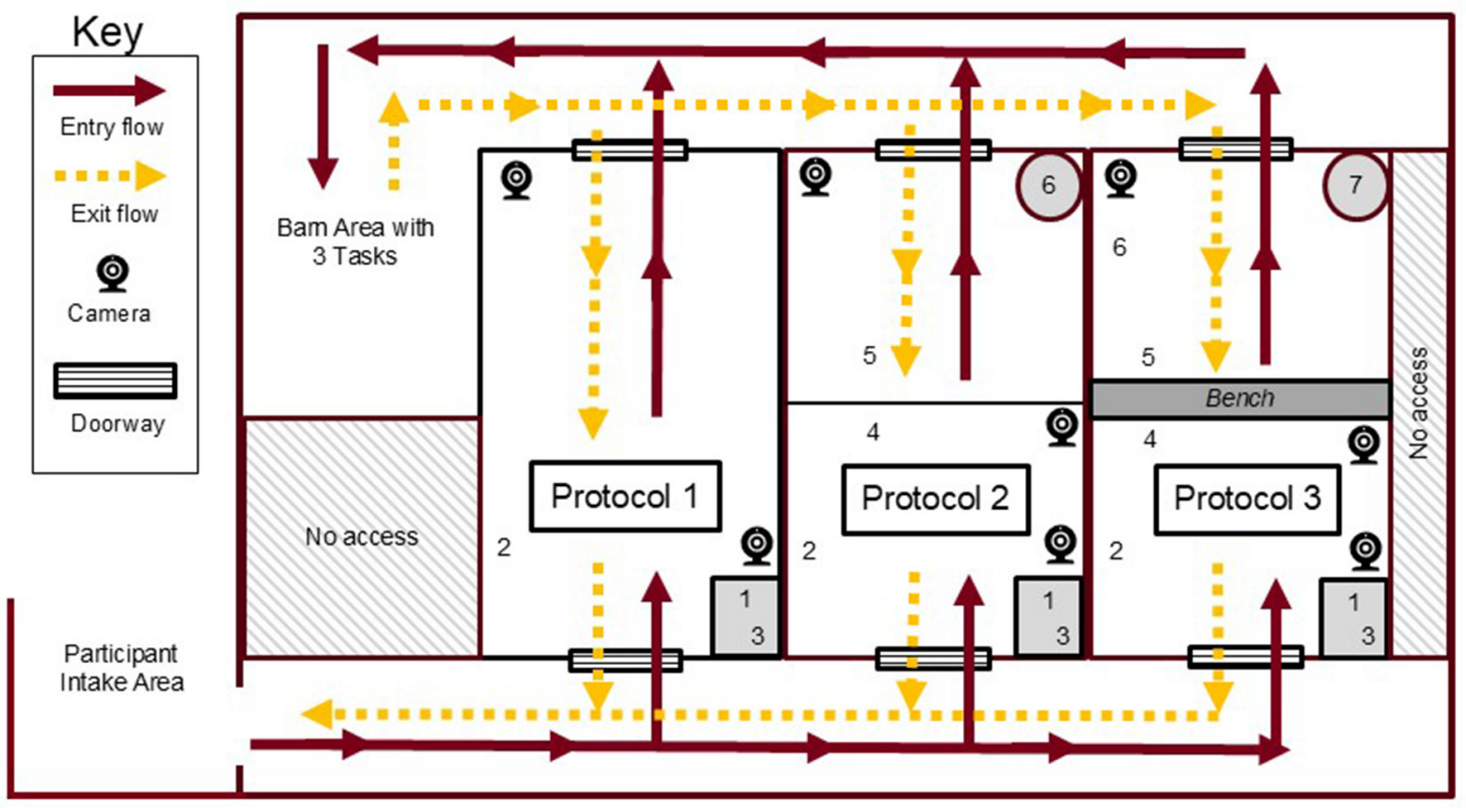
Plan view diagram picturing the three protocols, camera placement, directional flow, and number of steps performed in the simulated barn entry and exit research. Numbers within each protocol indicate what step and the location within the entry or exit it should be performed, as described in Table 1.

The experimental setting was a laboratory space on a university campus (Figure 1). The mock entryways were designated as Protocol 1 (P1), Protocol 2 (P2) and Protocol 3 (P3, Figure 2), situated inside a laboratory space. Each space was a simulated livestock barn entry (17), separating the exterior environment from the area that would house animals. Required biosecure entry and exit procedures for each protocol are described in Table 1, which included varying structural features and consumables such as boots, coats, and hand sanitizer. Each space measured 3.05 m by 1.41 m. The protocols used reflected common practices used in livestock and poultry production industries (9, 18).

**Table 1:** Assigned biosecure protocols for each simulated entry which was then reversed to exit the space.

**Step**	**Protocol 1 (P1)**	**Protocol 2 (P2)**	**Protocol 3 (P3)**
1	Sign and date the logbook		
2	Remove any personal outerwear and hang it on the clothing hooks. Remove and leave any removable personal belongings on the table		
3	Leave your phone in the storage area or seal your phone in a plastic bag to keep on your person		
4	–	Remove your footwear and leave it on the “dirty” side of the entryway, designated by a line on the floor/bench or Line of Separation	
5	–	Step over the line to the “clean” side, placing your feet in the barn footwear provided for you. Do not let your feet touch the floor on the clean side prior to putting on the boots.	Sit on the bench and swing your legs and feet over to the “clean” side, placing your feet in the barn footwear provided for you. Do not let your feet touch the floor on the clean side prior to putting on the boots.
6	–	Clean your hands with hand sanitizer located near the barn door.	Put on the overcoat provided on the “clean” side of the bench.
7	–	–	Clean your hands with hand sanitizer located near the barn door.
Total number of steps	3	6	7

A participant intake area was set up outside of the simulation areas (Figure 2). In the intake area, participants received entry instructions for a protocol and returned to the intake area to receive instructions for the next protocol. The simulated barn area consisted of a laboratory bench with three tasks laid out for each participant as a temporary distraction akin to work or activities within the barn, between the entry and exit processes. The three tasks differed for each entry and took approximately 5 min to complete in total. Instructions for the tasks were provided inside the barn area. Examples of tasks included completing a 24-piece puzzle, drawing a picture, or stacking blocks. These tasks were not evaluated.

Eight cameras were used to record data with placement indicated in Figure 2. Smart motion cameras (Model E892DD, Lorex, Linthicum, MD) were placed at ceiling height on the wall of each door to capture activity in each space. A third camera was included for protocols 2 and 3 on the floor at the LOS (a line on the floor for protocol 2 and bench for protocol 3) to capture activity specific to the LOS. All videos were recorded and archived using a network video recorder (Model N861, Lorex, Linthicum, MD).

### 2.2 Participants

Participants were recruited from within the university, were required to be 18 years of age or older and be a currently enrolled student (undergraduate, graduate, and professional), staff or faculty member. Recruitment efforts focused primarily on agricultural-focused departments, though other departmental affiliations were welcome to participate. They registered online using Qualtrics (Provo, Utah) and submitted demographic information including age, gender, and department affiliation.

Appointments for participants were scheduled one at a time for 45 min to eliminate congestion in the simulation area and to allow appropriate cleaning and disinfection of the simulation areas and supplies between participants.

Upon arrival at the simulation area, participants read and signed a consent form indicating they understood they would be video recorded for purposes of research and they would be engaging in physical activity.

Face masks were worn by all participants per COVID-19 guidelines at the University of Minnesota at the time of the experiment. Participants wore masks while executing entry and exit procedures.

Participants were compensated for their time.

### 2.3 Procedure

There were two rounds of data collection.

In round 1, all participants were randomly assigned an initial protocol and then completed the remaining protocols sequentially (e.g., P2, P3, P1). As shown in Table 1, the initial three steps of signing and dating a logbook, removing outerwear and handling of cell phones were common among protocols. Protocol 2 added three additional steps (6 total) where a participant also removed footwear, crossed the LOS while donning barn footwear, before sanitizing hands. Protocol 3 added four additional steps (7 total) to Protocol 1 such that the participant also removed footwear and crossed the LOS via a bench to don barn footwear, put on an overcoat and then sanitized hands before entering the simulated barn area. Ideally, handwashing stations would be available in a barn entry. For this simulation, plumbing was not available to offer handwashing stations in each entry and exit. This can be a similar scenario in older barns, particular weather climates and smaller operations, but alternatives like hand sanitizers can be provided. These protocols, as written, should not be adopted verbatim. In the barn environment, more frequent hand-cleaning should be considered at the LOS where risk is the greatest.

In round 1, the Protocols were delivered to a participant in one of three modalities (Methods): listen, read, or watch. Listening consisted of a researcher reading the assigned protocol aloud only one time to the individual participant. Reading meant the participant read the protocol to themselves, taking a reasonable amount of time, as necessary. Watching the protocol consisted of viewing a narrated, animated video (Doodly.com) up to 1 min and 38 s in length. For all modalities, if the participant asked questions, the researcher replied, “do the best you can,” or “do what you think is correct.” Participant instructions were to open the exterior door (Figure 1), pass through the entry completing the prescribed entry procedures and then use the barn door, opposite to the exterior door, to enter the simulated animal area. Participant instructions were to exit though the barn door, and eventually the exterior door using the reverse of the entry procedures.

After a period of 6–8 weeks, participants were invited to a follow-up session (Round 2). Participation was optional. Participants were again randomly assigned an initial protocol number to begin and then worked their way through the remaining protocols sequentially until completing all three. Entry protocols were not repeated to the participants, thus relying on recall of procedures. However, they were reminded of the total number of procedural steps of each entry (Table 1).

### 2.4 Measurements

Experience level was self-assessed on a 5-point Likert scale (21) by each participant during the first intake process. This question asked, “how much information do you know about biosecurity in animal agriculture?” This was intended to assess knowledge of biosecurity, rather than physical participation in biosecure practices.

Entry and exit errors were tallied by a single researcher using video recordings. For this purpose, an error was defined as a step performed incorrectly, out of sequence as delivered in the biosecurity procedure, or not completed. A procedural step performed incorrectly or incompletely was considered an action error. An action performed out of sequence was considered a sequence error. In some cases, steps two and three (Table 1) were optional according to the personal items worn and carried on the individual. Observations that did not link explicitly to the procedures but had implications for biosecurity were also counted. These include closing and latching entry and exit doors, buttoning a lab coat, and touching surfaces for balance.

Time for entry procedures was recorded and began when the simulated exterior door was opened. The time stopped when a participant closed the simulated barn door leading to the animal area or were no longer in the camera frame if they neglected to completely close the barn door. Recording time for exit procedures was reversed, beginning when the barn-side door was first opened until the exterior door was fully closed or the participant was no longer in the video frame. Time was recorded to the nearest second.

### 2.5 Statistical analysis

In this comparative study, an individual participant was considered an experimental unit. The participant was randomly assigned an educational delivery treatment (Method), and an initial protocol (Initial). Each participant was then exposed to all three protocols in round 1 and for those that returned, in round 2. For every participant and protocol combination, the category for Initial was recorded as IP (Initial Protocol) or NIP (Not Initial Protocol).

Response variables recorded were the entry time and exit time and total number of entry errors and exit errors of each protocol. Entry time and exit time (Time) in seconds were considered continuous and modeled using a linear mixed-effects regression. The number of entry or exit errors (Errors) were regarded as discrete and modeled using a mixed-effects Poisson regression.

The association between response and treatment variables was determined through a sequential Type I analysis of variance on the regression models. Treatment variables were added to the models in the following sequence: Round, Protocol, Method, Protocol × Method, Start, Start × Protocol, and Experience. This specific sequence was chosen to test the unique contribution of each variable after accounting for the preceding ones. A variable was retained in the model if its addition resulted in a significant improvement in the model's fit (*P* ≤ 0.05). The results of this analysis were summarized in an association matrix.

To quantify differences between treatments when a significant association was found, the final regression models were used. For Time, a linear mixed-effects model was used to determine the estimated mean difference between groups. For Errors, a Poisson mixed-effects model was used to determine the log-rate differences, which were then back transformed to incidence rate ratios to describe the relative difference in errors.

All analyses were completed using R (version 4.2.1) through RStudio (19). A significant difference was established at *P* ≤ 0.05. The *anova* function from the *faraway* package was utilized to build the association matrix. The *lmer* and *glmer* functions from the *lme4* package were used to fit the mixed-effects linear and Poisson regression models, respectively.

## 3 Results

In total, 59 participants completed round 1 of the trial. More students (34) participated in the study than university employees (11 faculty, 14 staff). Genders were split with more females participating than males (35 and 24, respectively). The mean age was 33 years old with a range of 18–71 years. The random assignments of education method and starting protocol are listed in Table 2 and resulted in an unbalanced experiment.

**Table 2:** Randomized distribution of participants' learning method (Method) and initial protocol (Initial) when participating in a biosecure entry and exit simulation.

**Method**	**Initial protocol**			
	**1**	**2**	**3**	**Total**
Listen	6	7	8	21
Read	6	5	6	17
Watch	9	8	4	21
Total	21	20	18	59

Round two had 33 returning participants, again with more students (20) than employees (four faculty, nine staff). Two sample independent proportions tests revealed the three University affiliation groups were equally represented in both rounds of this study. The mean age was 32 years with an age range of 18–69.

While the range of experience with biosecurity varied, few participants considered themselves very or extremely knowledgeable about biosecurity practices (Table 3).

**Table 3:** Self-assessed level of biosecurity knowledge on a Likert scale by individuals participating in a biosecure entry and exit simulation.

**Round**	**Level of biosecurity knowledge** ^*^					
	**1**	**2**	**3**	**4**	**5**	**Mean**
Round 1	13	19	16	9	2	2.5
Round 2	6	9	9	7	1	2.6

^*^Likert scale; 1= not knowledgeable at all, 2 = slightly knowledgeable, 3 = moderately knowledgeable, 4 = very knowledgeable, 5 = extremely knowledgeable.

### 3.1 Error types

Tables 4, 5 shows the types and counts of errors for each protocol during entry and exit, acknowledging whether the protocol was the protocol (initial protocol; IP), or not (non-initial protocol; NIP).

**Table 4:** The count of entry errors for protocols and additional observed errors for three different protocols, 4–6 weeks apart (round 1 or round 2) and whether the protocol was the participants' initial protocol (IP) or non-initial protocol (NIP).

**Entry**	**Protocol 1**				**Protocol 2**				**Protocol 3**			
	**Round 1**		**Round 2**		**Round 1**		**Round 2**		**Round 1**		**Round 2**	
	**IP**	**NIP**	**IP**	**NIP**	**IP**	**NIP**	**IP**	**NIP**	**IP**	**NIP**	**IP**	**NIP**
Log	0	0	0	0	1	0	0	0	0	1	0	0
Outerwear	1	1	1	3	3	2	2	3	2	2	1	5
Phone	0	0	0	0	0	0	0	0	0	0	1	0
Footwear	–	–	–	–	2	1	0	0	0	0	0	0
Boots	–	–	–	–	4	2	0	1	1	1	0	1
Lab coat	–	–	–	–	–	–	–	–	0	0	0	0
Sanitizer	–	–	–	–	0	0	0	0	0	0	0	1
Sequence	13	16	5	19	16	22	13	12	9	23	11	20
Total errors^*^	14	17	6	22	26	27	15	16	12	27	13	27
*P*-value action vs. sequence errors	0.07	0.06	0.17	0.06	0.0001	0.001	0.05	0.0002	0.001	0.02	0.002	0.0001
**Additional observed errors**												
Close external door	0	0	0	0	5	1	0	0	8	18	6	10
Close barn door	3	7	0	0	2	1	1	2	3	6	4	4
Lab coat not buttoned	–	–	–	–	–	–	–	–	28	8	15	6
1 hand wall touch	–	–	–	–	14	14	8	5	15	36	7	20
2 hand wall touch	–	–	–	–	14	1	3	6	1	45	1	23

^*^Sum of errors per protocol provided to participants. Does not include additional observed errors.

**Table 5:** The count of exit errors for protocols and additional errors for three different protocols, 4–6 weeks apart (round 1 or round 2) and whether the protocol was the participants' initial protocol (IP) or non-initial protocol (NIP).

**Exit**	**Protocol 1**				**Protocol 2**				**Protocol 3**			
	**Round 1**		**Round 2**		**Round 1**		**Round 2**		**Round 1**		**Round 2**	
	**IP**	**NIP**	**IP**	**NIP**	**IP**	**NIP**	**IP**	**NIP**	**IP**	**NIP**	**IP**	**NIP**
Sanitizer	–	–	–	–	0	2	2	1	0	1	1	2
Lab coat	–	–	–	–	–	–	–	–	2	0	2	5
Boots	–	–	–	–	4	7	1	1	1	3	0	2
Footwear	–	–	–	–	2	2	1	3	0	0	0	0
Phone	0	1	0	0	0	0	0	0	0	0	2	0
Outerwear	0	0	0	0	0	1	0	0	1	4	0	0
Log	0	1	0	0	3	1	1	0	1	3	0	0
Sequence	13	23	4	20	14	25	9	11	12	36	11	27
Total errors^*^	13	25	4	20	23	38	14	16	17	47	16	36
*P*-value action vs. sequence errors	0.4	0.4	0.6	0.3	0.0001	0.0001	0.0001	0.0001	0.0001	0.0001	0.0001	0.0001
**Additional observed errors**												
Close barn door	1	2	0	0	0	1	0	1	2	3	5	3
Close external door	4	6	0	0	2	3	1	1	7	16	6	7
1 hand wall touch	–	–	–	–	11	24	9	13	14	36	7	15
2 hand wall touch	–	–	–	–	2	9	9	0	14	36	6	13

^*^Sum of errors per protocol provided to participants. Does not include additional observed errors.

Errors were differentiated as either an action error or a sequence error. Sequence errors occurred more often than action errors for protocols 2 and 3 during entry and exit (Tables 4, 5; *P* ≤ 0.0001, *P* ≤ 0.0001, respectively). Protocol 1 entry and exit action errors were not statistically different. Predominant action errors included not visibly removing all outerwear (i.e., hats, jacket, jewelry/watch). When crossing the LOS, there were 18 entries and exits where individuals placed stocking or bare feet on the floor of the clean side of the room after contact with the dirty side floor.

Additional noteworthy observations occurred that were not part of the explicit protocols. Many participants placed one or both hands on the wall during protocol 2, for balance when donning boots. While all the doors for entry and exit to the simulated areas were the same, the greater count of doors not fully closed in protocol 3 could be attributed to a poor latching mechanism on the external door. Additionally, more than 61% of round 1 participants did not button the lab coat completely during protocol 3 (*P* ≤ 0.0001), and only 29% of round 2 participants fully buttoned their lab coat during the same protocol (*P* ≤ 0.0003).

### 3.2 Predictors for errors and time

**Figure 3:**
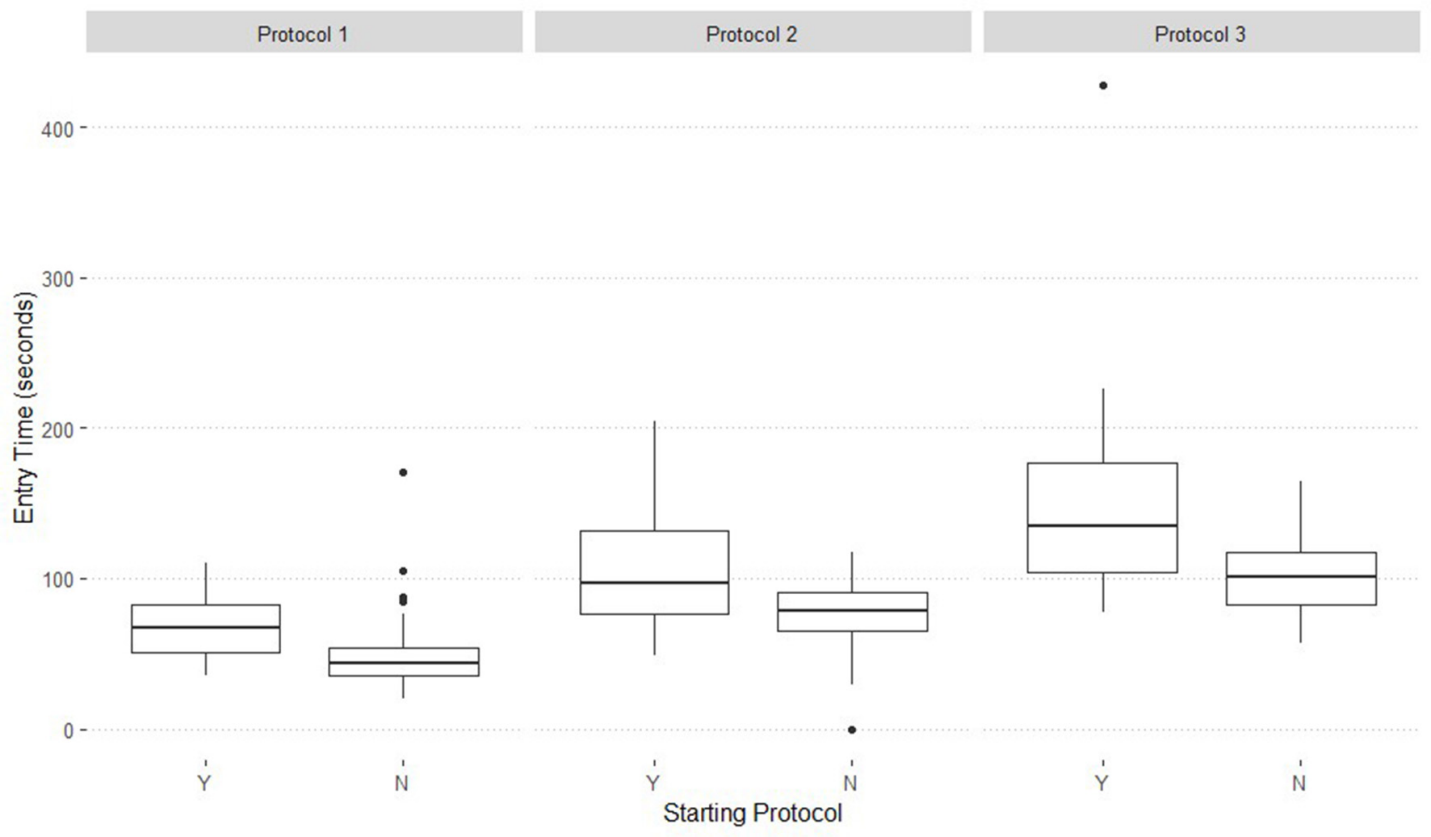
Comparison of entry time by starting protocol (Y) compared to non-starting protocol (N) within a Protocol when individuals participated in a biosecure entry and exit simulation.

Table 6 shows the association matrix linking the responses (entry errors, exit errors, entry time, exit time) to the predictors (round, protocol, method, and start). Education method and its interaction with protocol was not significant. It was also determined that Experience did not have an effect on explaining Entry or Exit Errors. The number of Errors committed during a protocol was not impacted by a participant's starting protocol. However, the association matrix shows the Start protocol and the interaction of Protocol and Start are significant predictors for Entry Time (Figure 3). Exit Time was not different between those that started with a particular protocol and those that started somewhere else.

**Table 6:** Association matrix of round, protocol, learning method (method), interaction of protocol and method, initial protocol (initial) and the interaction of protocol and start as predictors of time and errors.

**Variable**	**Round**	**Protocol**	**Method**	**Protocol × Method**	**Initial**	**Protocol × Start**
Entry errors	✓	×	×	×	×	×
Exit errors	✓	✓	×	×	×	×
Entry time	✓	✓	×	×	✓	✓
Exit time	✓	✓	×	×	×	×

✓indicates variable should be retained for the respective model. × indicates variable should not be retained for the respective model.

Table 7 presents the association matrix previously mentioned, which provides the coefficients to predict Error and Time responses for significant treatments, relative to the average response for the base case (intercept).

**Table 7:** Intercepts, coefficients of predictors of time responses, and log estimates of predictors on error responses.

**Variable**	**Intercept**	**Round_2_**	**Protocol_2_**	**Protocol_3_**	**Initial**	**Protocol_2_ × Initial**	**Protocol_3_ × Initial**
**Entry errors**							
Intercept (α)	−0.406	–	–	–	–	–	–
Coefficient (β^	–	0.317	–	–	–	–	–
$e^{\hat{\beta }}$	–	1.37	–	–	–	–	–
**Exit errors**							
Intercept (α)	−0.471	–	–	–	–	–	–
Coefficient (β^)	–	0.175	0.384	0.626	–	–	–
$e^{\hat{\beta }}$	–	1.191	1.468	1.871	–	–	–
**Entry time**							
Intercept (β_0_)	–	–	–	–	53.582	–	–
Coefficient (β_1_)	–	−10.482	–	–	–	–	–
Coefficient (β_2_)	–	–	25.704	–	–	–	–
Coefficient (β_3_)	–	–	–	51.838	–	–	–
Coefficient (β_4_)	–	–	–	–	16.779	–	–
Coefficient (β_5_)	–	–	–	–	–	16.055	–
Coefficient (β_6_)	–	–	–	–	–	–	30.336
**Exit time**							
Intercept (α)	32.99	–	–	–	–	–	–
Coefficient (β^)	–	−5.172	33.934	53.571	–	–	–

$e^{\hat{\beta }}$ = log transformed estimate of Errors committed compared with round 1, Protocol 1. β_4_ = mean difference for P1 between those who started with P1 and those that did not. β_5_ = mean difference for P2 between those who started with P2 and those that did not. β_6_ = mean difference for P3 between those who start with P3 and those that did not.

**Figure 4:**
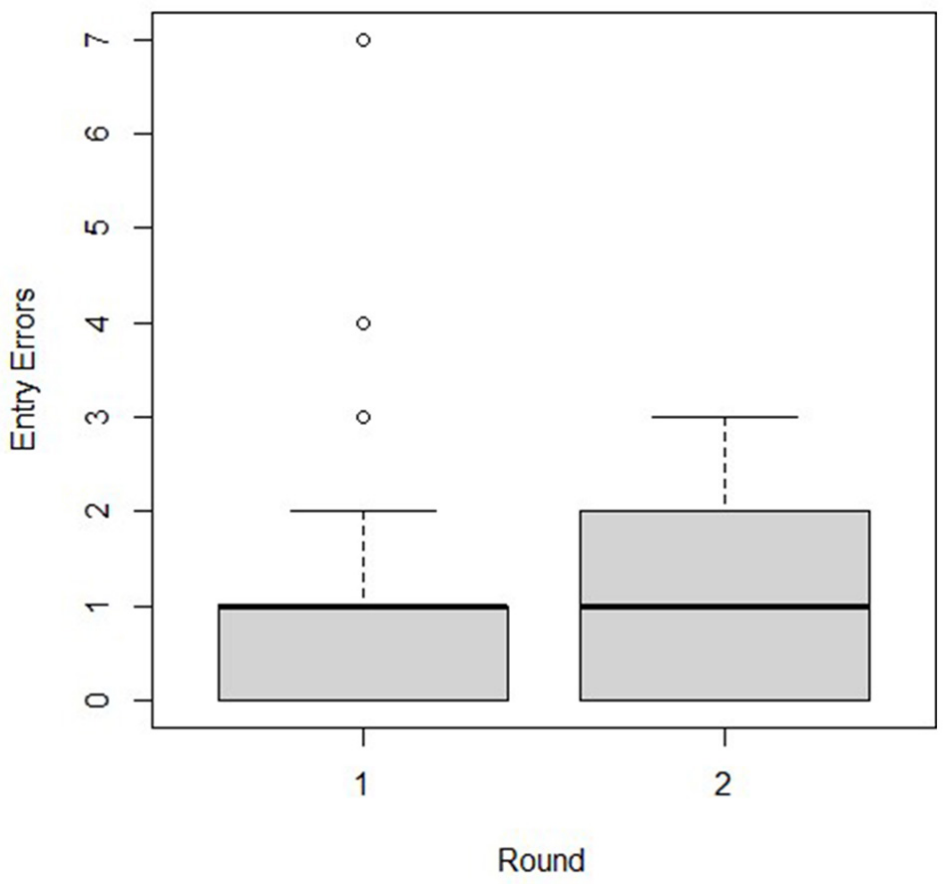
Entry errors by round when individuals participated in a biosecure entry and exit simulation.


The optimal model for Entry Errors had one predictor, Round, which had two levels (round 1 or round 2; Figure 4). The final model was:

$$\begin{array}{l} log\hat{\left(EntryErrors\right)}=\;-0.406\;+\;0.317\;\cdot 1\;\left(Round=2\right) \end{array}$$

The participants on average made 1.37 times more Entry Errors (*P* ≤ 0.02) during round 2 than the average number of errors committed during round 1.

**Figure 5:**
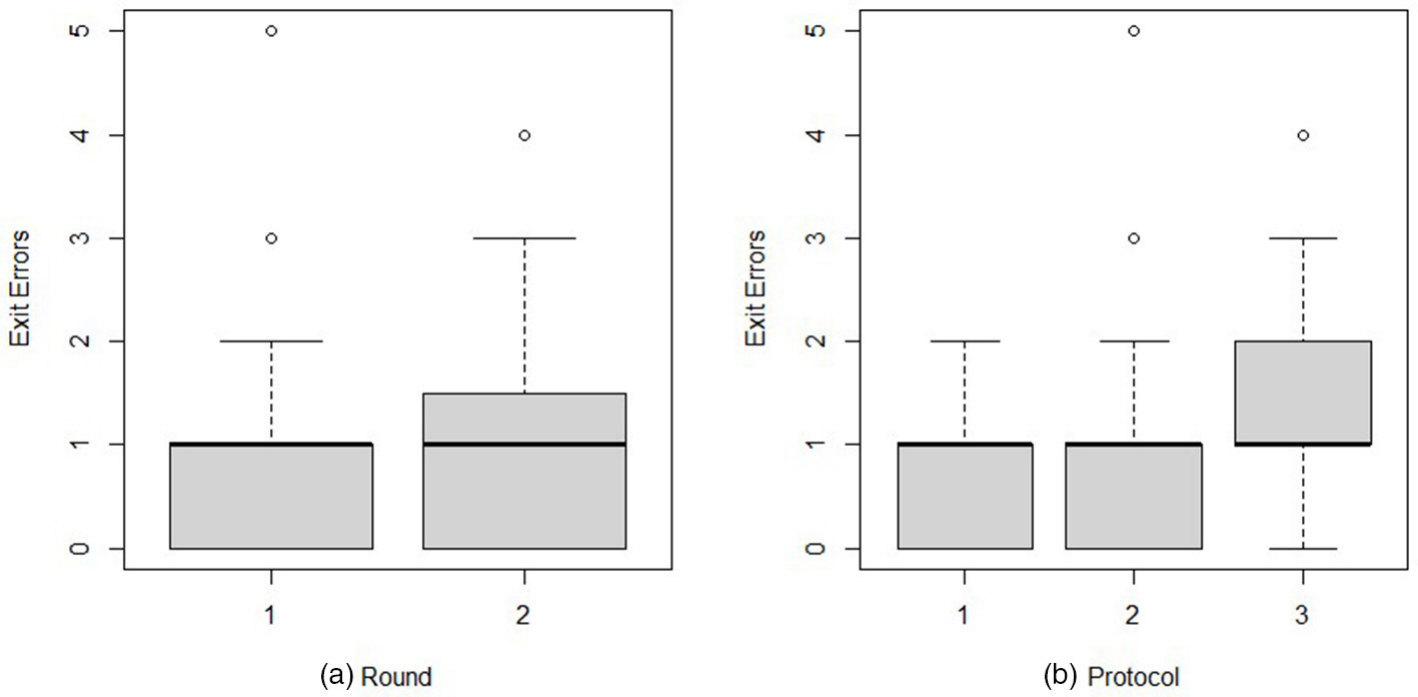
Exit errors by round **(a)** and protocol **(b)** when individuals participated in a biosecure entry and exit simulation.


The best model for predicting Exit Errors involves Round and Protocol (Figure 5). Using the coefficients in the model output, the final model was:

$$\begin{array}{r} log\hat{\left(ExitErrors\right)}=\;-0.471\;+\;0.175\;\cdot 1\;\left(Round=2\right)\\ \;+\;0.384\;\cdot 1\;\left(Protocol=2\right)\\ \;+\;0.626\;\cdot 1\;\left(Protocol=\;3\right) \end{array}$$

In round 2 on average, participants made 1.2 times more Exit Errors than the average of round 1 (*P* ≤ 0.17). Participants made 1.5 times more Errors on average with Protocol 2 than Protocol 1 (*P* ≤ 0.02). This factor becomes 1.9 for Protocol 3 compared with Protocol 1 (*P* ≤ 0.001).

**Figure 6:**
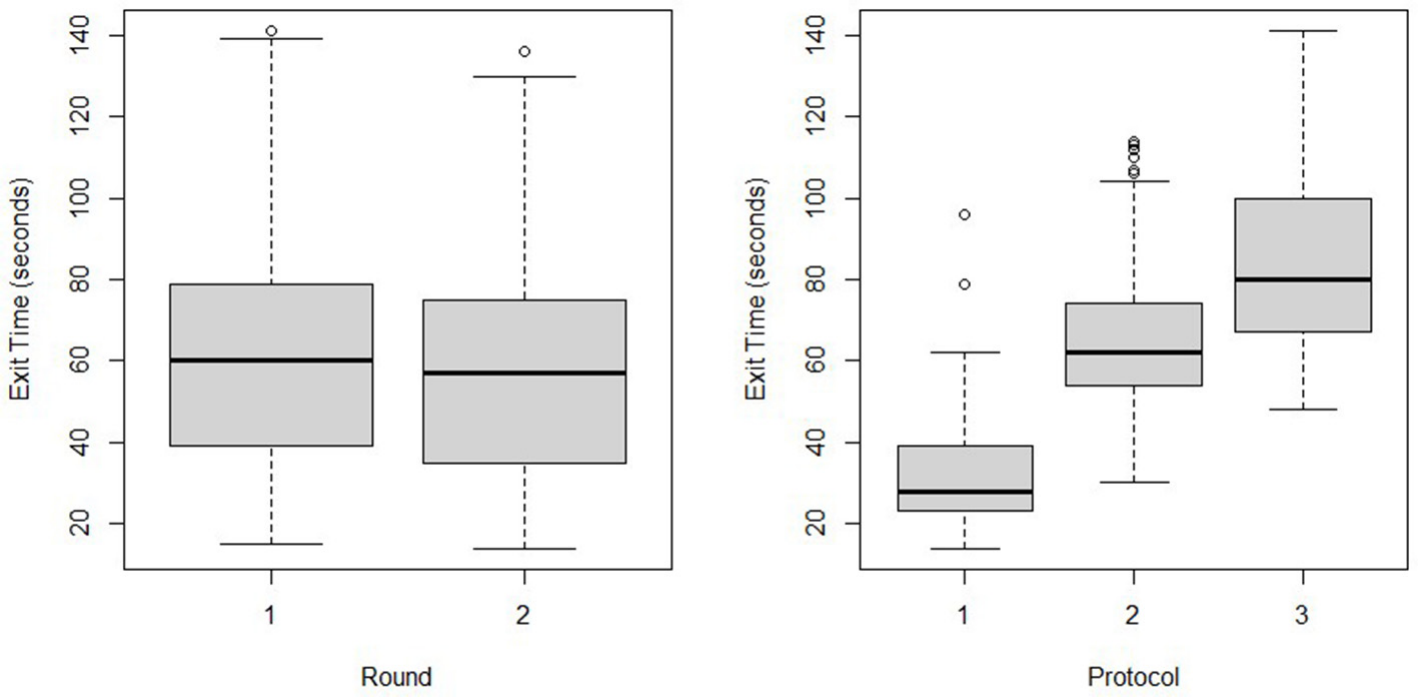
Exit time by round **(a)** and protocol **(b)** when individuals participated in a biosecure entry and exit simulation.


The best fit model for Entry Time considers Round, Protocol, the Start protocol, and the interaction of Start × Protocol (Figures 3, 6).

$$\begin{array}{l} \hat{Time}=\;53.582-10.482\;\cdot 1\;(Round=2)\\ +\;25.704\cdot 1\;(Protocol=2)+51.838\;(Protocol=3)\\ +\;16.779\;\cdot 1(Initial{=}^{\prime }IP^{\prime })+16.055\;(Initial{=}^{\prime }IP\prime )\cdot \\ 1\;(Protocol=2)\\ +\;30.336\cdot 1\;(Initial=\prime IP\prime )\;\cdot 1\;(Protocol=3) \end{array}$$

Participants that started with Protocol 1 took on average 17 s longer to complete the protocol than those who did not start with Protocol 1 (*P* ≤ 0.001). The average time difference for Protocol 2 between those who started there and those that did not was 33 s (*P* ≤ 0.001). Participants that started with Protocol 3 took an average of 47 s longer to complete the protocol than those that did not start with Protocol 3 (*P* ≤ 0.001).

For Exit Time, the best model includes Protocol and Round (Figure 6) resulting in the final model:


$$\begin{array}{r} \hat{ExitTime}=\;32.99-5.172\;\cdot 1\;\left(Round=2\right)\\ +\;33.934\;\cdot 1\;\left(Protocol=2\right)\\ +\;53.571\;\cdot 1\;\left(Protocol=\;3\right) \end{array}$$

Within any Protocol, people in round 2 on average had a lower Exit Time by 5.17 s. In either Round, participants spent an additional 34 s completing Protocol 2 than Protocol 1 (*P* ≤ 0.001) and 54 s more with Protocol 3 than Protocol 1 (*P* ≤ 0.001).

## 4 Discussion

We hypothesized that the method by which a participant received instructions would influence the number of entry and exit errors in the three simulated biosecurity protocols. While Merrill et al. (16) learned the use of graphics improved learning of biosecurity risk over reading or listening, our results showed no difference between the three education methods used for teaching biosecurity protocols. It was observed by researchers felt facemasks impeded instruction delivery for participants that were randomly selected for the listening method. Regardless of this, it did not impact on the number of errors. While not documented, could be a bias, or a weakness of the study—look of confusion on participants face, etc. but, if they were unfamiliar with biosecurity, they still may have look confused.

Since each protocol had a different number of steps to complete (i.e., 3, 6, or 7), it was expected that the amount of time needed to complete each protocol would differ. The results quantify these differences, which can be practically useful to show the correlation between the number of steps in a protocol and the effort required to complete them. Also, potentially related to a participant's comfort, their first protocol took more time than their peers that did not start with that protocol. This also illustrates that some experience (completing their first protocol) likely helped build knowledge and confidence for their subsequent protocols.

The effect of round on errors demonstrated how biosecurity compliance can change over time. This suggests training needs to happen more frequently for all that enter a biosecure premises. All visitors should be included in this training as our research showed experience was not a predictor of errors or the time needed to complete a protocol. The simulated spaces did not have protocol steps listed inside, such as a written protocol or a poster depicting expectations. Providing such resources may decrease errors on subsequent visits. This is a consideration that could be evaluated.

Entry and exit times were faster on average in round 2, suggesting participants relied on their previous experience, or comfort with the requirements, to successfully execute the protocol steps. However, the reduction in entry time during round 2 could also be attributed to the higher number of entry errors in round 2, suggesting errors were uncompleted steps, rather than out of sequence.

The sample size for round 2 was only 54% of round 1. Even though there was a financial incentive, potential explanations for choosing not to participate could include scheduling conflicts, perception of the importance of the study, self-selection, or feeling they did not successfully complete the tasks during the first round.

Throughout the experiment, over half of the errors were sequence errors vs. action errors. From a biosecurity perspective, the sequence while crossing the LOS is likely more important to risk reduction than the sequence of events before, or the sequence of events after. However, there were 18 LOS-specific errors, or approximately 19% of all errors, during entries for Protocols 2 and 3. Most of these errors involved dirty soles meeting the clean floor.

The lack of removal of outerwear could also be considered a LOS-error. The camera placement made it challenging to see whether jewelry and watches were on the participant and subsequently removed. There are also differing opinions on whether ball caps, vests and stocking caps are outerwear or not. In this project, these items were considered outerwear. The handling of cell phones was a separate step in the protocol. Most participants placed their phone in a bag regardless of whether they kept the phone with them or kept the cell phone on their person. These observations demonstrate the power of explicit instructions while recognizing there is a risk of overwhelming the individual with an increasing number of required steps.

Operational biosecurity supports structural biosecurity, and vice versa. The poor-latching door in protocol 3 was not intentional, but reflective of a real-world situation where a door may not function properly. For protocols 2 and 3, placing hands on walls for balance was necessary for the participant's personal safety. This situation exemplifies a value in demarking clean and dirty areas beyond the floor. In this type of situation, a farm may designate the wall as “dirty” with hand-cleaning events before and after the LOS.

The simulated entryways within the laboratory could not capture all the nuances of a real-world setting. Starting indoors influenced the type and amount of outerwear worn by participants. We provided appropriately sized boots and lab coats for each participant (with an extra set of boots in an alternative size), which are not always present in every entryway on every farm. Entryways were kept clear of debris, signage, and equipment to prevent participant distraction and ensure easy navigation in each space. Racicot et al.'s (11) paper tracked real world entries (434) and exits (449) of poultry farm workers, visitors and owners. The protocols in the study were not uniform between the eight selected sites because of farm-specific protocols already in place. Overall, the paper described 44 different errors made which justify explicit instructions for each entry and exit, which our results also support.

This project did not examine demonstration as a form of training, nor did it have written or graphical instructions in the entryway. The instructions within the entryway may prove useful for return visits with no additional education. Future research projects may consider additional training methods, extended return periods, or more variation in entryway setups and protocols between visits.

## 5 Summary

Our study objective was to evaluate education methods for biosecure barn entry and exit protocols by evaluating the number of errors, length of time to complete and how it affected compliance over time using biosecure protocols common in poultry industries Our results show that the number of steps required in a biosecure procedure and the length of time between visits are more effective predictors of biosecure entry and exit errors than the method used to teach the protocols.

## Data Availability

The raw data supporting the conclusions of this article will be made available by the authors, without undue reservation.
